# Advances in performance, more benefits... the perspectives of rapid response teams

**DOI:** 10.5935/0103-507X.20160048

**Published:** 2016

**Authors:** Marcio Manozzo Boniatti

**Affiliations:** 1 Hospital de Clínicas de Porto Alegre - Porto Alegre (RS), Brazil.

Rapid response teams (RRT) emerged in 1990 with the goals of improving the identification of ward patients with clinical deterioration and offering, based on this identification, early intervention.^(1-3)^ An RRT is activated according to previously defined triggers, traditionally vital signs, by themselves or as part of aggregated scores, other clinical changes, such as seizures, or even by a subjective criterion of concern about a patient. Once activated, the RRT evaluates the patient within five minutes, preferably, and defines the required procedures, such as fluid administration, antibiotic initiation, ventilatory support, and transfer to the intensive care unit (ICU). The presence of RRT in hospitals has been strongly suggested by organizations such as the Joint Commission and Institute for Healthcare Improvement.^(4,5)^ This suggestion is based on the possible benefit of providing early critical care to patients with deterioration, combined with evidence from "before and after" studies of cardiac arrest reduction.^(1,6)^ With the wide spread of rapid response systems, a constant increase has been observed in publications related to multiple elements of this model.^(1)^ Recently, other potential benefits, in addition to strategies to improve the performance of RRT, have been described.

In this edition of the *Revista Brasileira de Terapia Intensiva*, Mezzaroba et al. present a retrospective cohort study on the implementation of RRTs led by intensivists in university hospitals.^(7)^ Although the RRT performance in this study has been restricted to 12 daytime hours, the initiative has produced the following quality criteria:^(8)^ the "dose" delivered by the RRT was 102 calls per 1,000 admissions in the first year, with a median of two minutes for the arrival of the RRT at the bedside. Even with the decline in the number of calls in the following years, the "dose" was still well above the recommended minimum rate (25 per 1,000).^(9)^ Although the authors highlight the risk factors for hospital mortality, the performance characteristics of the RRT itself are the most relevant data. The subjective criteria of concern about the patient was the main trigger used to activate the RRT, reinforcing its importance in increasing the low sensitivity of objective criteria.^(10,11)^ In addition, the RRT had, among its responsibilities, to visit critical patients who remained in the ward daily. This is consistent with broader RRT activity, which has been suggested to include, for example, proactive visits and follow-up of patients discharged from the ICU.^(12,13)^ The retrospective design and the decrease in the number of calls are possible limitations of this study. The authors describe that the decrease in calls may be due to the implementation of daily visits; however, it is very likely that professional and/or cultural barriers have contributed.

Among the new perspectives of rapid response systems is the suggestion of using unexpected death as an outcome to evaluate the effectiveness of RRT.^(9,14)^ Unexpected death is defined as death without prior definition of treatment limitation. Patients with incurable diseases and/or in terminal stages are often in wards, usually under end of life palliative care. The performance evaluation of an RRT in preventing death or cardiac arrest should not include these patients. Therefore, unexpected hospital mortality seems to be a more appropriate outcome. The effect of the introduction of an RRT is more pronounced when this outcome replaces general hospital mortality.^(9,14)^

Another unexpected benefit that is often reported is the participation of RRT in end of life care.^(15)^ The activation an RRT is potentially a sentinel event for the recognition of end-of-life patients, resulting in subsequent enhancement of the discussion about adoption of unique palliative care.^(16,17)^ Several studies have demonstrated an increase in "do not resuscitate" requests and better documentation of comfort measures after the introduction of RRTs in the hospital.^(17-19)^ In 7 to 14% of RRT calls, a new definition of treatment limitation is initiated during or after the service.^(20,21)^ These findings exhibit the failure of identification of these patients in the ward.^(22)^ RRT have been shown to be an alternative for better identification and handling of these patients.^(15,16)^

The use of electronic algorithms that generate risk stratification in real time is another breakthrough that has been gaining prominence.^(23,24)^ These algorithms use electronic medical data, such as vital signs and laboratory and demographic data (for example, age and prior hospitalization in the ICU), to generate a direct electronic alert to RRT, without the need for calls. In addition, the algorithm is recalculated in real time for each new laboratory variable or registered vital sign.^(23,24)^ This advance may represent a solution to one of the most substantial barriers to successful deployment of RRT - call delays.^(25)^ Taking into account the increased mortality associated with call delays,^(25,26)^ it is possible that earlier notification could enable better outcomes in patients assisted by RRT. In addition to electronic algorithms, continuous monitoring systems with contact-free sensors have been tested to enhance acquisition of vital signs with promising results.^(27)^

The premise of the rapid response system is to offer care, by expert professionals, to critical patients anywhere in the hospital. The RRT, implemented based on patient's needs, without the usual geographical area restriction, has allowed specialists to leave the four walls of the ICU. Critical illness does not start when the patient enters the ICU, nor does it end when the patient is discharged from the ICU.^(28)^ This idea of continuum critical care guides the activity of RRT and reinforces the importance of leaving the confinement of the ICU. The discussion about the need for hospitals to adopt this security strategy focused on the patient seems to be over. Our challenge is qualifying this performance.
